# Editorial: Management of Sjögren's Syndrome

**DOI:** 10.3389/fmed.2021.836182

**Published:** 2022-01-13

**Authors:** Alessia Alunno, Clio P. Mavragani, Francesco Carubbi

**Affiliations:** ^1^Internal Medicine and Nephrology Unit, Department of Life, Health and Environmental Sciences, University of L'Aquila, L'Aquila, Italy; ^2^Department of Physiology and Joint Rheumatology Program, School of Medicine, National and Kapodistrian University of Athens, Athens, Greece; ^3^Department of Medicine, ASL1 Avezzano Sulmona L'Aquila, L'Aquila, Italy

**Keywords:** Sjögren's syndrome, COVID-19, ultrasonography, therapy, biomarkers, biologic therapies

Primary Sjögren's syndrome (pSS) is a systemic autoimmune disease characterized by chronic inflammation of exocrine glands and a clinical picture dominated by signs and symptoms of mucosal dryness (1). Owing to its systemic nature, up to 75% of patients with pSS experience extra-glandular manifestations ranging from mild arthralgias to life threatening cryoglobulinemic vasculitis (1). Among these subjects, 5% can develop B-cell lymphoma that is the most severe complication of pSS (2). Historically, the management of pSS has relied on expert opinion and the off-label use of drugs known to be effective in similar diseases such as systemic lupus erythematosus and rheumatoid arthritis (3). The first set of recommendations on the management of pSS has been formulated very recently by the European Alliance of Associations for Rheumatology (EULAR) (4) but several needs remain still unmet requiring additional research. In particular, despite showing a certain degree of efficacy on reducing disease activity and improving glandular secretion, most biologic agents investigated in recent years did not show promising results on patient-reported symptoms and fatigue. Since these items are stronger predictors of health-related quality of life in pSS compared to systemic involvement (5), it is of paramount importance to reflect on data from clinical trials and real-life settings collected so far and build on the successes and failures of the various compounds.

In our Research Topic, Vitali et al. reviewed the existing literature about pSS management with particular focus on the issues behind the failure of clinical trials investigating new therapeutic agents and the need of reliable biomarkers to distinguish the different clinical phenotypes. In addition, Chowdhury et al. summarized the evidence from recent clinical trials regarding the effects of biologic agents in modulating local inflammation and improving salivary gland function in the setting of pSS.

A discrepancy between the burden of symptoms reported by patients and the objective quantification of secretory function is often observed in pSS and adds a layer of complexity to the management of glandular manifestations (6). On this basis, Lackner et al. published an original research article in our Research Topic investigating a newly developed assessment tool, the pSS Quality of Life Questionnaire (QoL), for quantifying symptoms of dryness in comparison with the EULAR SS Patient Reported Index (ESSPRI) and the objective measurements of salivary and lacrimal flow. According to their findings, a correlation between the objective measurements of salivary gland function and the PSS-QoL but not the ESSPRI was observed, putting forward the hypothesis that different patient reported outcomes may allow to distinguish distinct groups of pSS patients and therefore have important implications in daily practice and clinical trials.

In recent years, salivary gland ultrasonography (SGUS) gained increasing interest in the field of pSS as a useful tool to better characterize patient subsets and ultimately drive therapeutic decisions along with clinical and serological features. In our Research Topic two articles focused on this area. Zabotti et al. reported the results of a Europe-wide reliability exercise to compare two SGUS scores assessing the reliability among sonographers with different levels of experience. They observed not only that both scores reliably identify SG abnormalities in pSS patients but also that adequate training allows less-expert operators to reliably assess pSS-SG by US. In addition, Zandonella-Callegher et al. compared the clinical and serological picture of pSS patients with normal or pathological SGUS and demonstrated that the former display a milder, more often seronegative phenotype, with a lesser degree of inflammation detected at SG biopsy.

Finally, the severe acute respiratory syndrome coronavirus 2 (SARS-CoV-2) pandemic affected the management of people with rheumatic and musculoskeletal diseases (RMDs), including those with pSS. We published in this Research Topic the first study addressing the personal experience of pSS patients during SARS-CoV-2 outbreak, demonstrating that disease activity and patient reported symptoms significantly worsened during the period of rheumatology services closure. Furthermore, we observed a variable impact of different work settings on the type of symptom worsening Carubbi et al.

In summary, our Research Topic demonstrated that the field of pSS management is rapidly evolving and that precision medicine should be pursued also in pSS as in other RMDs to optimize the therapeutic potential of currently available and upcoming compounds. Unfortunately, at the moment the evidence allowing to identify patients who could benefit from different targeted treatments and at different disease stage is scarce. For this purpose, clinical, serological, histological and ultrasonographic biomarkers should be identified to ultimately improve the prognosis and quality of life and people suffering from this disease (7).

## Author Contributions

All authors listed have made a substantial, direct, and intellectual contribution to the work and approved it for publication.

## Conflict of Interest

The authors declare that the research was conducted in the absence of any commercial or financial relationships that could be construed as a potential conflict of interest.

## Publisher's Note

All claims expressed in this article are solely those of the authors and do not necessarily represent those of their affiliated organizations, or those of the publisher, the editors and the reviewers. Any product that may be evaluated in this article, or claim that may be made by its manufacturer, is not guaranteed or endorsed by the publisher.
